# Eye-tracking technology and the dynamics of natural gaze behavior in sports: an update 2016–2022

**DOI:** 10.3389/fpsyg.2023.1130051

**Published:** 2023-06-08

**Authors:** Ralf Kredel, Julia Hernandez, Ernst-Joachim Hossner, Stephan Zahno

**Affiliations:** Institute of Sport Science, University of Bern, Bern, Switzerland

**Keywords:** gaze behavior, visual search, eye-tracking, eye movements, sports

## Abstract

Updating and complementing a previous review on eye-tracking technology and the dynamics of natural gaze behavior in sports, this short review focuses on the progress concerning researched sports tasks, applied methods of gaze data collection and analysis as well as derived gaze measures for the time interval of 2016–2022. To that end, a systematic review according to the PRISMA guidelines was conducted, searching Web of Science, PubMed Central, SPORTDiscus, and ScienceDirect for the keywords: eye tracking, gaze behavio*r, eye movement, and visual search. Thirty-one studies were identified for the review. On the one hand, a generally increased research interest and a wider area of researched sports with a particular increase in official’s gaze behavior were diagnosed. On the other hand, a general lack of progress concerning sample sizes, amounts of trials, employed eye-tracking technology and gaze analysis procedures must be acknowledged. Nevertheless, first attempts to automated gaze-cue-allocations (GCA) in mobile eye-tracking studies were seen, potentially enhancing objectivity, and alleviating the burden of manual workload inherently associated with conventional gaze analyses. Reinforcing the claims of the previous review, this review concludes by describing four distinct technological approaches to automating GCA, some of which are specifically suited to tackle the validity and generalizability issues associated with the current limitations of mobile eye-tracking studies on natural gaze behavior in sports.

## 1. Introduction

In 2017, Kredel et al. (2017) published a review on eye-tracking technology and the dynamics of natural gaze behavior in sports that the scientific community has quite well received. This systematic review covered 40 years of previous research, beginning with the trend-setting publication on visual search activity in sports by Bard and Fleury (1976) and ending with the comparison of elite and non-elite tennis players’ gaze behaviors by Murray and Hunfalvay (2016). Based on 60 included studies, the authors reasoned that sports-related eye-tracking research seemed to strive for ecologically valid test settings (i.e., concerning viewing conditions and response modes) and experimental control along with high measurement accuracy (i.e., controlled test conditions with high-sample-rate eye-trackers linked to algorithmic analyses). To meet both demands, the authors suggested the integration of robust mobile eye-trackers in motion-capture systems whilst, at the same time, advising researchers to carefully weigh arguments concerning the fundamental trade-off between laboratory and field research. In any case, further advancements in mobile eye-tracking methodology seemed advisable to allow for more significant amounts of gaze data to increase the explanatory power of the inferred results (*cf.* also Orquin and Holmqvist, 2018).

Given the highly dynamic developments in sports technology over recent years, the present review aims to complement the data reported by Kredel et al. (2017) by updating the analysis for the time interval of 2016–2022, thereby especially focusing on the progress concerning researched sports tasks, applied methods of gaze data collection and analysis as well as derived gaze measures.

## 2. Methods

In line with the PRISMA guidelines (Shamseer et al., 2015), a systematic review was conducted by searching the electronic databases Web of Science, PubMed Central, SPORTDiscus, and ScienceDirect for the keywords: eye tracking, gaze behavio*r, eye movement, and visual search (February 2022). Studies on natural dynamic visual behavior with gaze assignments on at least two different areas of interest (AOIs) written in the English language and published in peer-reviewed journals between 2016 and 2022 were included, whilst studies on perceptual training, using occlusion paradigms or without the collection of eye-tracking data were excluded. After removing duplicates from the initially identified 491 articles, 387 studies were screened by two independent raters leading to the exclusion of 260 additional studies that were not sports-related (99), not based on eye-tracking (90), actually not peer-reviewed (53) or not dealing with natural dynamic gaze behavior (18). Assessing the full text of the remaining 127 records for eligibility resulted in a further exclusion of 96 articles that were not sports-related (41), conducted without pre-defined AOIs (24), focused on non-natural or static gaze behavior (19), actually not employing eye-tracking (11) or researching perceptual learning (1). The remaining 31 articles were finally included in the review.

## 3. Results

In the following paragraphs, the review results will be summarized and contrasted with the review conducted by Kredel et al. (2017).

### 3.1. Publications

The 31 included studies are characterized in Table 1 by author names and year of publication, researched sport, sample size, visualization condition, viewing perspective, required motor response, number of analyzed trials, applied eye-tracker type and sample rate (ET), gaze-cue allocation method (GCA), number of pre-defined areas of interest (NAOI) as well as derived gaze measures, namely fixation durations (FD), number of fixations (NF), saccades (SA), viewing times (VT) and gaze dynamics, either not related (DN) or related (DE) to a specific event. In purely quantitative terms, the number of scientific publications on natural gaze behavior in sports has increased significantly in recent years (from 11 in 2007–2011 over 20 in 2012–2016 to 28 in 2017–2021).

**Table 1:** Overview of sports-related eye-tracking studies (2016–2022).

Publication	Task					Gaze analysis				Gaze measures					
**Author(s)/Year**	**Sport**	***N***	**Condition**	**View**	**Response**	**Trials**	**ET**	**GCA**	**NAOI**	**FD**	**NF**	**SA**	**VT**	**DN**	**DE**
Spitz et al. (2016)	Soccer (R)	39/39	Lab	1st	Artificial	20/20	s/120	Algorithmic	4	x	x	–	–	–	–
Zeuwts et al. (2016)	Cycling	12/13	Lab+Field	1st	Natural	550 m/700 m	m/50(30)	Manual	5	–	–	–	–	–	–
Chia et al. (2017)	Badminton	24/24	Field	1st	Natural	27.6/30	m/30(−)	Manual	6	x	x	–	x	–	–
Decroix et al. (2017)	Slalom Skiing	11/27	Field	1st	Natural	17/17	m/50(25)	Manual	8	x	x	–	x	–	–
Schnyder et al. (2017)	Soccer (R)	6/6	Field	1st	Natural	36/36	m/120(60)	Manual	7	x	x	–	–	–	x
Seifert et al. (2017)	Climbing	18/18	Field	1st	Natural	1 route	m/60(30)	Manual	40	x	x	–	–	x	–
Vansteenkiste et al. (2017)	Cycling	27/34	Field	1st	Natural	550 m/700 m	m/30(−)	Manual	5	x	x	–	x	–	–
Brimmell et al. (2018)	Soccer	41/42	Lab	1st	Natural	2/2	m/30(−)	Manual	4	x	x	–	x	–	–
Del Campo et al. (2018)	Soccer (R)	22/22	Lab	1st	Natural	24/24	m/−	Manual	2	x	x	–	x	–	–
Goh et al. (2018)	Bowling	21/21	Field	1st	Natural	10/10	m/25(−)	Algorithmic	8	x	x	–	x	–	–
Hunfalvay and Murray (2018)	Tennis (WC)	32/32	Lab	1st	Natural	18/18	s/60	Algorithmic	5	x	x	–	–	–	x
Pizzera et al. (2018)	Gymnastic (J)	35/35	Lab	1st	Natural	19/21	s/300	Manual	5	x	x	–	–	–	–
Robertson et al. (2018)	Judo (C)	20/22	Lab	1st	Natural	2/24	m/30(−)	Manual	9	x	x	–	x	–	–
Sáenz-Moncaleano et al. (2018)	Tennis	21/21	Field	1st	Natural	40/40	m/60(−)	Manual	4	x	–	–	–	–	x
Van Maarseveen et al. (2018)	Basketball	13/13	Field	1st	Natural	12/36	m/24(−)	Manual	9	x	x	–	x	x	–
Kim et al. (2019)	Archery	14/14	Lab	1st	No	3/3	s/−	Algorithmic	5	x	x	x	–	–	x
Vila-Maldonado et al. (2019)	Volleyball	38/38	Lab	1st	Artificial	34/36	m/− (−)	Manual	8	x	x	–	x	–	–
Aksum et al. (2020)	Soccer	5/5	Field	1st	Natural	16 min	m/− (25)	Manual	4	x	–	–	x	–	–
Bickmann et al. (2020)	Soccer (E)	21/21	Lab	1st	Natural	1 match	s/120	Manual	9	x	x	–	–	–	–
Kato (2020)	Kendo	20/20	Field	1st	Natural	4/5 sess.	m/60(−)	Manual	6	x	x	–	x	–	–
Mitchell et al. (2020)	Climbing (C)	6/6	Field	1st	Artificial	12/12	m/60(−)	Manual	3	x	x	–	x	–	–
Shearer et al. (2020)	Team Sports	34/34	Lab	1st	Artificial	11/11	s/120	Manual	3	x	x	–	–	–	–
Babadi Aghakhanpour et al. (2021)	Fencing (R)	28/28	Lab	1st	Natural	50/50	m/60(−)	Manual	4	x	x	–	–	–	–
Esteves et al. (2021)	Basketball	10/10	Field	1st	Natural	50/50	m/60(−)	Manual	6	x	x	–	–	–	–
Klatt et al. (2021a)	Basketball (R)	8/9	Field	1st	Natural	1 match	m/200(120/30)	Manual	6	x	–	x	–	–	x
Klatt et al. (2021a,b)	Field Hockey	13/14	Field	1st	Natural	19.5/20	m/200(120/30)	Manual	6	x	–	–	–	–	x
Loiseau-Taupin et al. (2021)	Badminton	19/19	Field	1st	Natural	2 sets	m/200(120)	Manual	9	x	x	–	x	–	x
Rosker and Majcen Rosker (2021a)	Tennis	15/15	Field	1st	Natural	45/90	m/50(−)	Manual	25	x	–	–	–	–	x
Rosker and Majcen Rosker (2021b)	Tennis	17/17	Field	1st	Natural	60/∼90	m/50(−)	Manual	25	x	–	–	–	–	x
Krabben et al. (2022)	Judo	7/8	Field	1st	Natural	12.9/20	m/120(25)	Algorithmic	7	–	–	–	x	–	x
Van Biemen et al. (2022)	Soccer (R)	14/14	Field	1st	Natural	65.4/90 min	m/60/30(−)	Manual	6	x	x	–	x	–	x

The publications are specified by researched sport (R, referee; J, judge; WC, wheelchair; C, coach; E, e-sports), sample size (N: number of participants with gaze analysis/total number of participants), visualisation condition (Lab = slides/videos, Field = *in-situ* investigation), viewing perspective (1st = agent’s perspective, 3rd = agent on the field), required motor response (natural = natural action, artificial = verbal/button press/etc., no = just watching), number of trials (number of trials with gaze analysis/total number of trials), applied eye-tracker (ET, type and sample rate in Hz, m = mobile, s = stationary, − = not reported, in brackets: sample rate of scene camera), the gaze-cue allocation method (GCA, Manual = ratings, algo. = algorithmic, − = not reported), number of pre-defined areas of interest for gaze allocation (NAOI), as well as collected gaze measures (FD = fixation durations, NF = number of fixations, SA = saccades, VT = viewing times, DN = gaze dynamics not related to a specific event, DE = gaze dynamics related to a specific event, x = measured, − = not measured).

### 3.2. Task

Despite the recent increase in the total number of studies, the percentage of studies in game sports decreased from 87.1% (Kredel et al., 2017) to 61.3% in the current review, which not only reflects a broader range of researched sports but a marked focus on sports referees’ or judges’ gaze behavior (22.6%). The sample sizes have remained essentially constant (*M* = 20.7, SD = 10.3; compared to *M* = 20.6, SD = 12.4 for 1976–2016). However, it apparently has become standard practice to base gaze analyses not only on a fraction of the sample but on all participants. Overall, the total of 1′131 researched participants in 2016 increased to a total of 1′743 participants in 2022.

Regarding the experimental setting, a strong trend can be noticed toward ensuring the results’ ecological validity. Compared to Kredel et al. (2017), the percentage of studies in which gaze data was acquired under natural field conditions rose from 39.4 to 62.5%. Above, a natural viewing perspective was implemented in each of the more current studies, meaning that the scenery was presented to the participant as an evolving situation watched from a first-person perspective, contrary to 11.9% with a third-person perspective in the 2017 review. The same trend holds for the required motor response. The percentage of studies in which participants were asked to respond naturally to the presented situations (as opposed to button presses, verbal responses, etc.) increased from 60.7% for 1976–2016 to 83.9% for the recent 5-year interval.

**Figure 1:**
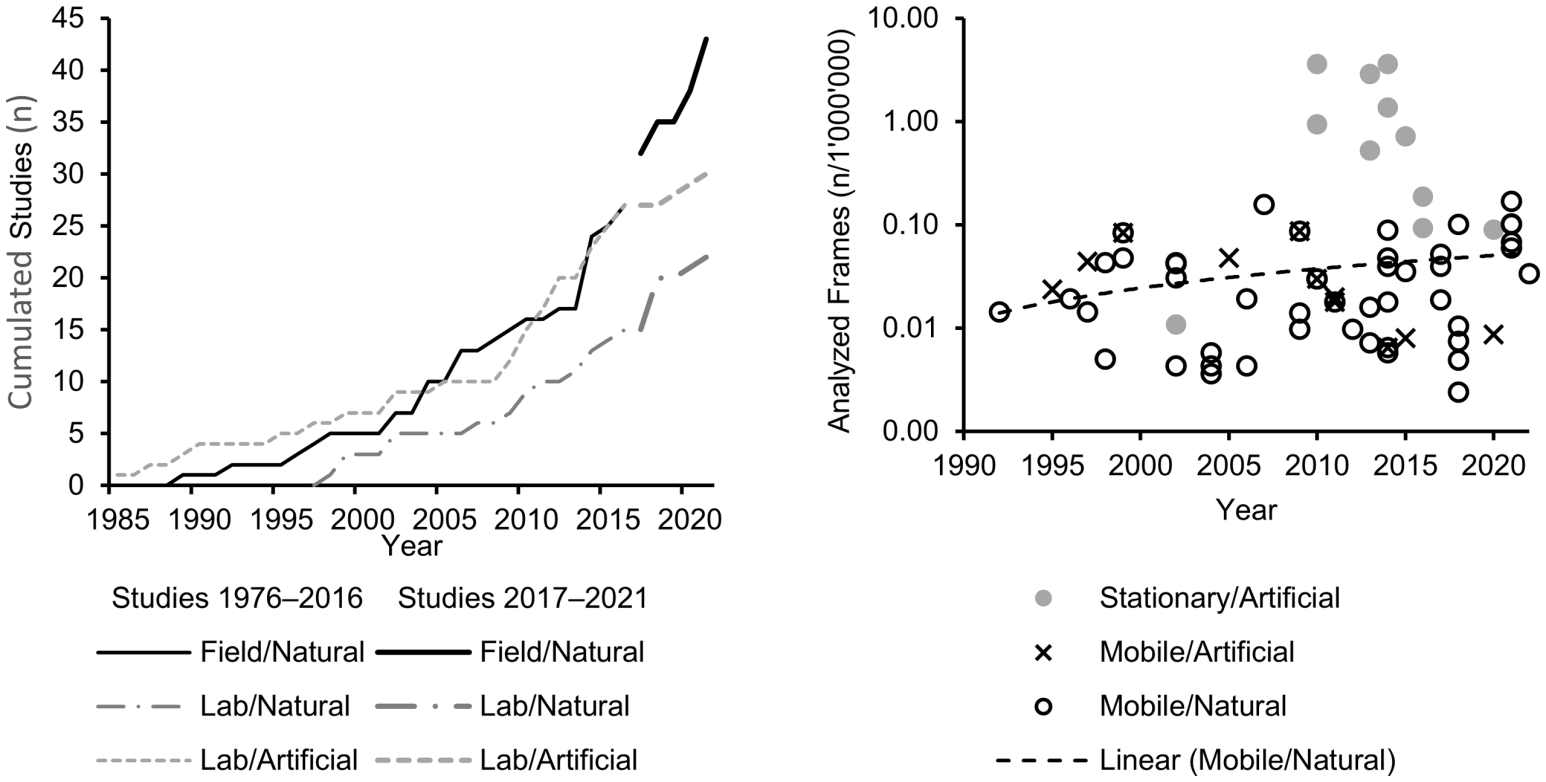
Cumulated number of studies published 1976–2021, assigned by the external validity to the categories “field/natural,” “lab/natural,” and “lab/artificial” (left) and estimated total numbers of analyzed frames per study as a function of year of publication (1993–2022), applied eye tracker (stationary vs. mobile), and required motor response (artificial vs. natural) (right, please note the log scale of the vertical axis).

The resulting tendency towards an emphasis on ecological validity is illustrated in summary in the left panel of Figure 1, in which the studies included in the 2017 and the 2022 review are grouped concerning the viewing conditions (field vs. lab) and response requirements (natural vs. artificial), respectively, and depicted in cumulation (thereby omitting a single publication in which no response was demanded as well as the field/artificial category that appeared only twice since 1976). Obviously, the portion of lab studies with artificial responses stagnates over recent years whilst the number of lab studies with natural responses, and more so, the number of field studies with natural responses has remarkably grown.

### 3.3. Gaze analysis

Compared to the review conducted by Kredel et al. (2017), the percentage of analyzed trials per participant slightly decreased from 82.3 to 79.5% and the number of total trials performed per participant considerably decreased from 41.5 (SD = 49.1) to 30 (SD = 22.9). Assuming no substantial prolongation of the single trials and given the substantial noise that gaze data is typically afflicted with, this decrease is a profound surprise.

On the other hand, the average sample rate raised in median values from 30 Hz to 60 Hz. This is partly because recent studies have used mobile eye-trackers with comparatively high-sample-rate cameras (up to 200 Hz). However, these numbers should be interpreted with caution as desirable reporting standards are still not obeyed in all cases (leading to, e.g., mixed-up eye and scene camera sample rates or sample rates not reported at all). When having a closer look at the used eye-trackers, it becomes evident that – despite the noticeable technological progress in mobile eye-tracking systems over the recent years (with, e.g., higher sample rates and video resolutions, multiple cameras per eye, and slippage correction) – most studies relied on comparably old technology with low specifications. Virtually never reported was a comprehensive set of data quality measures, as already proposed by Holmqvist et al. (2012), rendering further quality comparisons considerably more difficult, if not impossible.

The same absence of marked progress is revealed when considering the total number of analyzed frames per study – roughly estimated from the numbers of included participants, trials per participant, and eye-tracker sample rates as specified in the respective papers and assuming the analysis of a 2-s interval per trial (which, due to circumstances, can only be done for publications in which all these details are specified). The resulting numbers are depicted in the right panel of Figure 1 as a function of the year of publication, the applied eye-tracker, and the required motor response – again omitting a category that appeared only twice since 1976, namely stationary/natural. The use of stationary eye-trackers that feature high sample rates and thus remarkably raise the number of analyzed frames seems to mark a distinct episode lasting from 2010 to 2016. This turn away in more recent years can probably be best ascribed to the inevitable costs of stationary (and especially head-fixed) eye-tracking, namely, the reduction of ecological validity, as already discussed above. Likewise, no positive tendency is revealed for studies in which mobile eye-trackers came into operation, but artificial motor responses were required. In contrast, regarding mobile eye-tracking studies with natural responses, a slight increase can be noticed that not only refers to the total number of studies over consecutive 5-years intervals but also to the total number of analyzed frames. When calculating a linear trend for these investigations, this number increased from about 10′000 frames in 1990 to about 55′000 frames in 2022, corresponding to an annual increase of about 1′000 analyzed frames per study. However, compared to the depicted studies in which stationary eye-trackers allowed for more than 1 million analyzed frames per study, these numbers still fall short by orders of magnitude.

Regarding the number of AOIs reported in the papers included in the present review, the current gaze point was allocated to about eight predefined AOIs (*M* = 8.2, SD = 7.7), a number that pretty much matches the count over the previous time interval (*M* = 6.8, SD = 3.1). Details of gaze-cue allocation (GCA) were only elliptically reported and therefore remain difficult to interpret. Some studies (e.g., Sáenz-Moncaleano et al., 2018) seem to employ algorithmic event-detection algorithms (e.g., for fixations) first, and assign AOIs to events in a second step (leading to a significant reduction of manual work), whilst others (e.g., Van Maarseveen et al., 2018) perform a frame-by-frame GCA and classify fixations downstream based on these categorial allocations, obviously omitting detailed spatial characteristics of gaze locations. Some ignore these – perceptually relevant – event detections altogether or even redefine standard definitions (e.g., Loiseau-Taupin et al., 2021). Nevertheless, decent progress can be noticed concerning an algorithmic rather than a manual approach to GCA as – compared to the 8.3% reported by Kredel et al. (2017) – an algorithmic approach has been pursued in 16.1% of studies over more recent years. However, for three of the total five studies with an algorithmic GCA, the automation was at the expense of accepting a stationary collection of gaze data (Spitz et al., 2016; Hunfalvay and Murray, 2018; Kim et al., 2019). Still, the remaining two studies with a completely automated GCA (Goh et al., 2018; Krabben et al., 2022) represent significant progress in terms of algorithmic analyses of mobile eye-tracking data in sports-related settings (from 0% in 2017 to 8.0% more recently).

### 3.4. Gaze measures

Regarding derived gaze measures, Kredel et al. (2017) report no significant trends when comparing studies conducted either in 1976–2000 or 2001–2016. This picture seems to have considerably changed over more recent years. More precisely, comparing the past with the recent study pool, a remarkable drop in the percentage of studies is revealed that report overall viewing times (VT; from 88.3 to 48.4%; for a respective critique, see Orquin and Holmqvist, 2018) or gaze dynamics without relating the gaze behavior to specific events (DN; from 33.3 to 6.5%). Whilst this decrease can probably be best ascribed to the mostly nominal informative value of these two variables in dynamic settings, the additionally observed decrease in the analysis of saccades (SA; from 25.0 to 6.5%) sparks hope that also in sports-related studies the technological limitations of low sample-rate eye-tracking devices concerning saccade analyses slowly become respected or, at least, the established nomenclature is applied more consistently.

In contrast, two further gaze measures continue to be reported in most publications, namely the number of fixations (NF; from 85.0 to 74.2%) as well as fixation durations (FD; from 88.3 to 93.5%). Depending on their definition (*cf.* Hessels et al., 2018), these measures can be automatically derived from the raw gaze data in a relatively straightforward manner by applying event-detection algorithms (e.g., IVT, but refer to Andersson et al., 2017, for a performance evaluation of different algorithms and specific caveats before their application, especially in mobile settings).

The most pronounced increase, however, pertains to the calculation of variables that specify aspects of gaze dynamics related to specific events, which essentially means that GCA is aligned to specifics of temporal movement or situational unfolding (DE; from 23.3 to 35.5%). This trend is further underlined by the fact that this statement applies to the most recently conducted seven studies included in the present review, obviously reflecting a significant growth of scientific interest in event-related spatiotemporal dynamics of natural gaze behavior in sports, or – in more psychological terms – in the dynamic coupling of visual perception and action.

## 4. Discussion

Compared to the Kredel et al. (2017) review for the period from 1976–2016, the present update has revealed three relevant trends. First, a generally growing scientific interest in the field of research was observed. Second, aspects of ecological validity seem to become increasingly important, which is reflected by a growing fraction of studies in which gaze data is gathered under the natural, first-person viewing conditions of the field, and natural motor responses are required, which, in turn, calls for a preferable use of mobile eye-trackers that allow for (more or less) unconstrained behavior. Third, whilst the scientific interest in more general aspects of gaze behavior (e.g., overall viewing times) seems to diminish, a trend becomes evident towards focusing on gaze measures that specify spatiotemporal aspects of gaze dynamics by event-based GCA or higher-order classification into visual strategies.

In contrast, the progress with respect to the applied eye-tracking technology seems to be limited. Foremost, common reporting guidelines as proposed already 10 years ago and recently detailed by the mainstream eye-tracking community (Holmqvist et al., 2022) are still not comprehensively employed in sports-related studies. This, however, seems of utmost importance, especially due to the significant error potential associated to mobile eye-tracking in dynamic situations (Niehorster et al., 2020; Hooge et al., 2022). Without increasing the quality of the reporting and the underlying measurement process, the formulated quest for a larger amount of gaze data per study would fall considerably short, as just employing a faster eye-tracker cannot solve potential inherent data quality issues.

Nonetheless, the total number of analyzed frames per study does also not show a remarkable increase such that the 55′000 frames currently analyzed in mobile eye-tracking studies with natural motor responses still do not reach the standard for studies in which stationary eye-trackers were applied. One obvious solution to this problem – as already suggested by Kredel et al. (2017) – can be found in a more pronounced use of eye-tracking technology that allows for an algorithmic analysis of larger amounts of gathered gaze data. Applying appropriate event detection algorithms on high-sample-rate raw eye data, eye movements can be objectively and precisely classified (e.g., into fixations, saccades, or smooth pursuit). Beyond, concerning GCA, we currently identify four distinct approaches:

1. manual gaze/event-to-AOI-mapping using raw scene camera images,
2. automated gaze/event-to-AOI-mapping using pose estimation on scene camera images,
3. automated gaze/event-to-AOI-mapping using motion-capture integration and natural visual scenes,
4. automated gaze/event-to-AOI-mapping using motion-capture integration and artificially rendered visual scenes.

While (1) has been the *de facto* standard in mobile sports-related eye-tracking studies from the beginning, this approach has been refined by firstly employing event-detection algorithms on high-sample-rate eye-tracking data, and, in a second step, allocating these events to AOIs captured by a lower sample-rate scene camera. At the cost of losing cue-related scan paths, this leads to a significant reduction of workload while maintaining temporal precision for event-detection. Approach (2) can be seen as an extension to (1), which both seem particularly suited for unconstrained field studies. Employing algorithms for visual scene understanding (for a recent review on human pose estimation see Kumar et al., 2022), the manual AOI assignment in (1) is substituted by automated approaches in (2). While these two approaches dramatically economize the gaze analysis process and (2) also increase the objectivity of the GCA, two further advantages are specifically achieved by motion-capture integration, that is, (a) the possibility to add AOIs that do not appear in the currently analyzed video frame (e.g., the location where a tennis ball will be hit by the racket), and (b) a synchronized recording of the participant’s movements, which allows relating the algorithmic gaze analysis to action events (e.g., the moment of response initiation). While approach (3) emphasizes external validity by presenting natural visual scenes, where, for instance, real opponents’ limbs are motion-tracked, (4) employs artificially rendered visual scenes to maximize experimental control. By displaying either video content with previously identified trajectories of crucial AOIs or computer-generated visual scenes (e.g., leveraging game engines, where object trajectories are known *a priori*), both approaches allow a fully automated gaze-vector-based GCA as already proposed by Kredel et al. (2010, 2015). Both approaches could be scaled to constrained field research by using LPM- or IMU-based motion-capture, even if they seem particularly suited for constrained lab research. Employing head-mounted AR/VR displays with integrated eye-trackers can be seen as special cases for (3) or (4), having identical advantages, but currently still the apparent drawbacks of head-mounted high-mass and high-inertia devices for dynamic sports tasks.

To sum up, approaches as proposed in (2), (3) or (4) would allow for a substantial increase in analyzed gaze data per study without corrupting the ecological validity of test conditions and the internal validity of the measurements. This would not only enhance the generalizability of the obtained results; a respective increase would more so create conditions for a critical re-examination of findings on potential differences in gaze behavior that are overall accepted in literature but ultimately based on relatively small sample sizes. For instance, the general belief that expert athletes are distinguished by a comparatively “quiet” gaze behavior with fewer fixations of longer durations (*cf.* Mann et al., 2007; Gegenfurtner et al., 2011) – that has been questioned in recent reviews and meta-analyses (Klostermann and Moeinirad, 2020; Silva et al., 2022) – would be open to an empirical test when pursuing those algorithmic approaches. We thus would like to conclude that the application of reporting standards and reliable high-sample-rate mobile eye-trackers with algorithmic analysis of a more significant amount of gathered gaze data will be the decisive step forward in future research on the dynamics of natural gaze behavior in sports.

## Author contributions

All authors listed have made a substantial, direct, and intellectual contribution to the work and approved it for publication.

## Funding

Open access funding by University of Bern.

## Conflict of interest

The authors declare that the research was conducted in the absence of any commercial or financial relationships that could be construed as a potential conflict of interest.

## Publisher’s note

All claims expressed in this article are solely those of the authors and do not necessarily represent those of their affiliated organizations, or those of the publisher, the editors and the reviewers. Any product that may be evaluated in this article, or claim that may be made by its manufacturer, is not guaranteed or endorsed by the publisher.
